# Role of Perivascular Adipose Tissue-Derived Adiponectin in Vascular Homeostasis

**DOI:** 10.3390/cells10061485

**Published:** 2021-06-12

**Authors:** Adrian Sowka, Pawel Dobrzyn

**Affiliations:** Laboratory of Molecular Medical Biochemistry, Nencki Institute of Experimental Biology, Polish Academy of Sciences, 02-093 Warsaw, Poland; a.sowka@nencki.edu.pl

**Keywords:** endothelial cells, vascular smooth muscle cells, atherosclerosis, obesity, adipose tissue

## Abstract

Studies of adipose tissue biology have demonstrated that adipose tissue should be considered as both passive, energy-storing tissue and an endocrine organ because of the secretion of adipose-specific factors, called adipokines. Adiponectin is a well-described homeostatic adipokine with metabolic properties. It regulates whole-body energy status through the induction of fatty acid oxidation and glucose uptake. Adiponectin also has anti-inflammatory and antidiabetic properties, making it an interesting subject of biomedical studies. Perivascular adipose tissue (PVAT) is a fat depot that is conterminous to the vascular wall and acts on it in a paracrine manner through adipokine secretion. PVAT-derived adiponectin can act on the vascular wall through endothelial cells and vascular smooth muscle cells. The present review describes adiponectin’s structure, receptors, and main signaling pathways. We further discuss recent studies of the extent and nature of crosstalk between PVAT-derived adiponectin and endothelial cells, vascular smooth muscle cells, and atherosclerotic plaques. Furthermore, we argue whether adiponectin and its receptors may be considered putative therapeutic targets.

## 1. Introduction

The growing obesity epidemic, especially in Western countries, has prompted the need to discover novel therapeutic strategies for various conditions that are related to obesity, such as cardiovascular disease, which is the most prevalent cause of mortality and morbidity in developed countries [1]. The main feature of obesity is substantial adipose tissue volume expansion. Understanding the biological mechanisms by which adipose tissue expands became a compelling research area that led to the discovery that adipose tissue not only stores lipids in the form of triglycerides but also secretes various molecules, termed adipokines that exert effects on both adjacent and remotely located organs [2,3]. Perivascular adipose tissue (PVAT) is a particularly interesting fat depot because of its anatomical location. It surrounds nearly all blood vessels except the cerebral vasculature, suggesting a possible connection with vascular homeostasis. Indeed, PVAT was shown to play a significant role in both health and disease states through adipokine secretion, which has been extensively reviewed elsewhere [1,4]. One of the most abundant adipokines, adiponectin, is especially interesting when considering its unique structure [5] and pleiotropic actions on numerous cellular processes, such as lipid and glucose metabolism [6,7,8], insulin signal transduction [9], and inflammation [10]. The present review discusses recent advances in the research area of adiponectin signaling in PVAT and its influence on the vascular wall.

## 2. Perivascular Adipose Tissue: Structure and Function

The traditional view of the vascular wall structure comprises a three-layer model. The first layer, termed the tunica intima, consists of endothelial cells and has direct contact with blood. The second and thickest layer is the tunica media, which consists mainly of smooth muscle cells that regulate vascular tone through their ability to alternate contraction and relaxation events. The third layer, the tunica adventitia, consists of different cell types, primarily fibroblasts that play a mainly supportive role. In this three-layer model, PVAT is not considered a part of the vascular wall. For many years, PVAT was only seen as structural support for blood vessels. In traditional contraction studies, PVAT was removed because it was not suspected to play any notable role. This perception was reconsidered when PVAT was shown to influence aorta responsiveness in rats [11]. The important role of PVAT was further established when adipocyte-derived relaxing factors (ADRFs) were identified [12,13]. PVAT exhibits both a white adipose tissue (WAT) phenotype and a brown adipose tissue (BAT) phenotype, depending on its anatomical location. Brown adipose tissue derived its name from its macroscopic appearance, which is determined by its higher vascularization and thus more abundant blood supply. Brown adipose tissue characteristics also include more abundant mitochondria, multiple, small lipid droplets, a greater amount of cytoplasm, and the expression of uncoupling protein 1 (UCP1), which mediates mitochondrial non-shivering thermogenesis. White adipose tissue macroscopically appears lighter with less of a blood supply. White adipose tissue does not express UCP1. A typical WAT adipocyte contains one large lipid droplet, a peripherally located nucleus, and a smaller cytoplasm [14]. The existence of either type of adipocyte within PVAT differs, depending on the vessel’s anatomical localization. For example, the thoracic aorta in mice is surrounded by PVAT with more of a BAT-like phenotype [15]. In addition, thoracic PVAT in rats was shown to resemble BAT, while abdominal PVAT had more features that were specific to WAT [16]. PVAT, like every other adipose tissue, secretes cytokines, hormones, growth factors, and adipocyte-specific molecules, termed adipokines. Adipokines are involved in regulating whole-body energy status through food intake regulation (e.g., by leptin) and other processes, such as inflammation, glucose uptake, fatty acid oxidation, and reactive oxygen species formation (e.g., by leptin, adiponectin, visfatin, and resistin). Some of these molecules were also shown to act as vasoactive agents that induce vasodilation and vasoconstriction. Adipose tissue plays a beneficial role as long as adipokine levels with opposing properties remain in equilibrium. At the onset of metabolic conditions, such as obesity or type 2 diabetes, when this equilibrium is impaired in favor of proinflammatory or constriction-inducing agents, PVAT becomes dysfunctional and exerts detrimental effects with regard to vascular homeostasis. For example, it can promote endothelial cell dysfunction, recruit proinflammatory immune cells, and induce vascular smooth muscle cell (VSMC) proliferation. This dual nature of PVAT has thus been called a “double-edged sword” [1]. The dichotomous qualities of PVAT are further emphasized by the fact that human coronary arteries are embedded in abundant PVAT and are one of the most susceptible arteries to atherosclerosis, whereas murine coronary arteries are completely devoid of PVAT and develop atherosclerotic plaques in aortic stretches, such as the aortic arch [17]. Histological discrepancies between the anatomical localization of PVAT appear to be clinically significant because the abdominal aorta is more susceptible to aortic aneurysm development than the thoracic part of the aorta [17,18]. Genome-wide transcriptional studies revealed significant differences between PVAT that surrounds the area of the dilated aortic area (i.e., an area with an aneurysm) compared with non-dilated areas. Components of innate and adaptive immunity were overrepresented in PVAT adjacent to aneurysms, supporting the hypothesis that this type of vascular condition has an autoimmune nature, and a vast number of immune cells and cytokines originates from dysfunctional PVAT [19]. The human thoracic aorta is plausibly less susceptible to aneurysm development because of thoracic PVAT UCP1 expression, which sequesters fatty acids and thus prevents potential lipotoxic effects on the aortic wall [20].

## 3. Metabolic Functions of Adiponectin and Its Receptors

### 3.1. Structure of Adiponectin

Adiponectin is an adipocyte-derived adipokine that was first identified in blood plasma and differentiated 3T3-L1 cells [5,21]. Adiponectin contains 247 amino acids in mice and 244 amino acids in humans, and its predicted molecular weight is 30 kDa [5]. Adiponectin is encoded on chromosomes 3 and 16 in humans and mice, respectively [22]. Plasma levels of adiponectin are similar in humans and rodents and remain around μg/mL, which makes adiponectin nearly 1000-fold more abundant than insulin or leptin [23]. Adiponectin possesses structural features that resemble complement protein C1q, collagen VIII, and collagen X. Thus, it was initially named Acrp30 (adipocyte complement-related protein of 30 kDa) [5].

The adiponectin molecule consists of two domains: the C-terminal globular domain and the N-terminal collagen domain. In serum, adiponectin does not occur in monomeric form because of its fibrous domain, which allows it to oligomerize. Adiponectin forms trimers with a molecular weight around 67 kDa, two trimers that form hexamers of around 140 kDa, and multimers that consist of at least 18 monomers, termed high-molecular-weight (HMW) adiponectin [24]. The globular form of adiponectin, without a collagen-like domain, also exhibits activity [25]. Although adiponectin is mainly secreted by adipocytes, its plasma levels are inversely correlated with adipose tissue mass. Therefore, obese and diabetic patients exhibit lower circulating adiponectin [26]. The lack of adiponectin in mice led to greater susceptibility to high-fat diet-induced obesity and higher plasma tumor necrosis factor α levels, indicating its anti-inflammatory properties [27]. Furthermore, exogenous adiponectin administration improved insulin resistance and inhibited neointimal plaque formation in adiponectin knockout mice [28]. Direct antiatherogenic effects of adiponectin have only recently been reported. Adiponectin preferentially binds to oxidized low-density lipoprotein (oxLDL), thereby preventing its entry into the cell [29].

### 3.2. Adiponectin Receptors 1 and 2 in Regulation of Metabolism and Membrane Homeostasis

To date, three adiponectin receptors have been discovered: adiponectin receptor 1 (AdipoR1), AdipoR2 (both encoded by their respective genes) [30], and T-cadherin [31]. Mouse AdipoR1 is a 375-amino-acid protein with a molecular weight of 42.4 kDa, whereas AdipoR2 is a 311-amino-acid protein with a molecular weight of 35.4 kDa. AdipoR1 and AdipoR2 exhibit high homology between mice and humans (96.8% and 95.2%, respectively). The structures of both receptors are similar, with 66.7% identity between them. Although AdipoR1 and AdipoR2 have seven transmembrane domains, N-termini are intracellular, and C-termini are extracellular, which, combined with low sequence homology, makes them structurally distant from G-protein-coupled receptors (GPCRs) [32,33,34]. Both AdipoR1 and AdipoR2 have been implicated in the insulin signaling pathway and alterations of expression patterns of metabolic genes [35]. Although both receptors are expressed in many cell types, AdipoR1 expression is most abundant in skeletal muscles, and AdipoR2 expression is most abundant in hepatocytes. The activation of both receptors is connected with the activation of pathways that involve 5’-adenosine monophosphate-activated protein kinase (AMPK), p38 mitogen-activated protein kinase (MAPK), and peroxisome proliferator-activated receptor γ (PPARα), leading to increases in fatty acid oxidation and glucose uptake [6,30]. The muscle-specific disruption of AdipoR1 led to insulin resistance and a decrease in mitochondrial biogenesis [36]. Moreover, Yamauchi et al. [35] reported that AdipoR1 overexpression in the liver improved insulin resistance and decreased gluconeogenesis through the activation of AMPK, whereas AdipoR2 was more connected with PPARα activation. Controversial findings, however, have also been reported, in which two research groups independently discovered opposing effects of AdipoR1 and AdipoR2. Mice with AdipoR1 knockout exhibited glucose intolerance and an increase in body weight that was attributable to decreases in locomotion and overall energy expenditure. AdipoR2 knockout mice exhibited resistance to high-fat diet-induced obesity that was associated with higher energy expenditure and improved insulin signaling [37,38].

Although AdipoRs function in cells seems to be well established, a few novel insights into their biology have been recognized. AdipoR’s amino acids sequence shows homology with yeast IZH protein, which was shown to have an impact on plasma membrane fluidity through regulation of levels of structural membrane sterols [39]. It was also shown that human AdipoRs were able to mimic IZH proteins in yeast [40]. Similarities were also identified in Caenorhabditis elegans, where two AdipoRs homologs exist, and their disruption leads to membrane rigidification as a result of the accumulation of saturated fatty acids in plasma membranes [41]. This suggests that AdipoRs have an enzymatic activity that is independent of adiponectin, since C. elegans lacks adiponectin homolog [42]. Indeed, studies utilizing human cell lines showed that depletion of AdipoRs resulted in a higher accumulation of saturated fatty acids (SFAs) in plasma membranes, resulting in their rigidification [43]. Furthermore, AdipoR2 downregulation in human umbilical vein endothelial cells (HUVECs) and human embryonic kidney cells (HEK293) and subsequent treatment with SFA palmitate resulted in impaired the expression of desaturases as well as the induction of unfolded protein response. In addition, upon palmitate treatment, cells showed mitochondrial respiratory defects and poor viability [44]. Intriguingly, supplementation with mono- or polyunsaturated fatty acids, which are known to increase membrane fluidity, was able to revoke detrimental effects of AdipoR2 silencing [42,44]. These findings suggest that the primary function of AdipoRs may not be adiponectin binding but the regulation of membrane homeostasis. This implies that future studies on AdipoRs should be more focused on their adiponectin-independent activity, possibly by using in vivo models [42].

### 3.3. T-Cadherin Receptor

Another receptor for adiponectin is T-cadherin [31]. It belongs to a large group of proteins, called cadherins, with a different structure that is involved in cell–cell adhesion. In addition to T-cadherin, all members of the cadherin protein superfamily possess a transmembrane domain in their structure that ensures connections between extracellular signals and the intracellular environment. T-cadherin is anchored in the plasma membrane by a glycophosphatidylinositol anchor with no intracellular part. The mechanism by which a signal is transduced from the cell surface to the cytoplasm remains to be elucidated [45]. T-cadherin is known to be a key signal transducer between adiponectin and the cardiovascular system, and these two proteins often co-localize with each other in cardiovascular tissues, whereby adiponectin can exert its cardioprotective actions [46,47,48]. Beyond cardiovascular tissues, T-cadherin is also found in skeletal muscles and facilitates adiponectin accumulation in this tissue [49,50]. The regulation of T-cadherin expression is complex and not fully understood. Relationships have been reported between plasma adiponectin levels and mutations in the gene that encodes T-cadherin [51,52,53]. T-cadherin protein levels were also shown to be associated with adiponectin levels. In adiponectin knockout mice, T-cadherin levels were lower, and exogenous adiponectin administration increased T-cadherin levels [50]. Recent data suggest that adiponectin and T-cadherin, when bound together, accumulate in multivesicular bodies in endothelial cells and can be further secreted by exosomes [54]. This finding provides interesting insights into the mechanism by which adiponectin exerts its biological effects through T-cadherin because the former does not possess an intracellular domain. Recently, it has been demonstrated that the native adiponectin in mouse serum, which is primarily HMW adiponectin, strongly binds to the surface of cells expressing T-cadherin, but not AdipoRs [55]. It pinpoints the fact that the form of adiponectin (monomers, oligomers, or multimers) is a key feature when considering the preferential binding affinity of adiponectin. It should also be considered when conducting future experiments since the majority of studies utilize recombinant *E. coli-* derived adiponectin.

## 4. Intracellular Actions of Adiponectin

The binding of adiponectin on the cell surface results in several cellular effects. As mentioned above, although AdipoRs and GPCRs share common features, such as seven transmembrane domains, they are relatively distinctly related. Hence, the secondary messenger is not a G-protein. Nevertheless, they still need to be functionally bound to a protein that can relay extracellular adiponectin signals to induce adequate intracellular responses. Upon adiponectin binding to either AdipoR1 or AdipoR2, adaptor protein containing pleckstrin homology domain, phosphotyrosine binding domain, and leucine zipper motif (APPL1) are activated [56]. 

### 4.1. Adiponectin Mediated AMPK Activation

**Figure 1:**
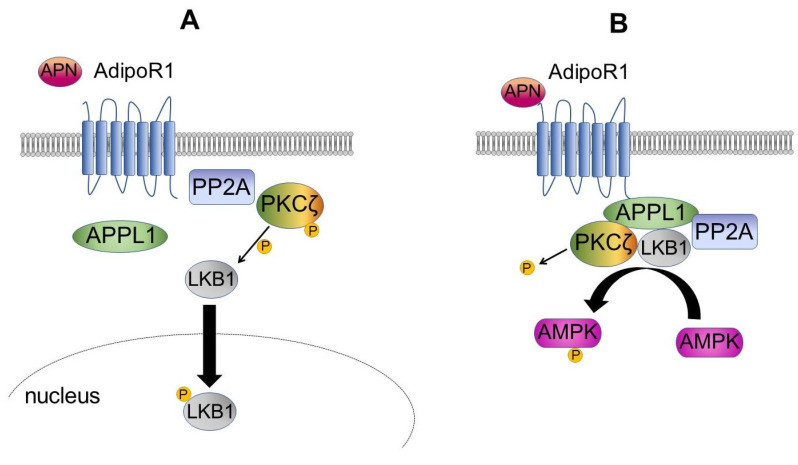
Mechanism of adiponectin-dependent AMPK activation. (**A**) In the non-stimulated state, APPL1 and protein phosphatase 2A (PP2A) do not interact. Protein kinase Cζ (PKCζ) phosphorylates liver kinase B 1 (LKB1) and induces its translocation to the nucleus. (**B**) Upon adiponectin (APN) binding, PKCζ is dephosphorylated by PP2A. Dephosphorylated PKCζ is no longer able to phosphorylate LKB1. PP2A also dephosphorylates LKB1, which results in LKB1 translocation to the cytoplasm. LKB1 phosphorylates AMP-activated protein kinase (AMPK) in the form of the AdipoR1/APPL1/PP2A/ PKCζ/LKB1 complex.

When adiponectin binds to AdipoR1, this activation results in the recruitment of protein phosphatase 2A (PP2A) and protein kinase Cζ (PKCζ) (Figure 1A). When AAPL1 is not activated, PKCζ phosphorylates liver kinase B1 (LKB1) at Ser307 and facilitates its translocation to the nucleus. However, after APPL1 activation, PP2A leads to PKCζ dephosphorylation (Figure 1B). Dephosphorylated PKCζ no longer has the ability to phosphorylate LKB1, and PP2A facilitates LKB1 dephosphorylation, resulting in the inhibition of LKB1 in the nucleus. When in the cytosol, LKB1 joins the AdipoR/APPL1/PP2A/PKCζ complex and phosphorylates AMPK (Figure 1B) [57,58]. 

Using a human cDNA screening in a yeast two-hybrid system approach, APPL1 was found to interact with Rab5, which is a small guanosine-5’-triphosphatase downstream of APPL1. It facilitates glucose transporter 4 (GLUT4)-containing vesicle translocation toward the plasma membrane and internalization, leading to an increase in glucose uptake [56]. 

### 4.2. Adiponectin Mediated p38 MAPK Activation

**Figure 2:**
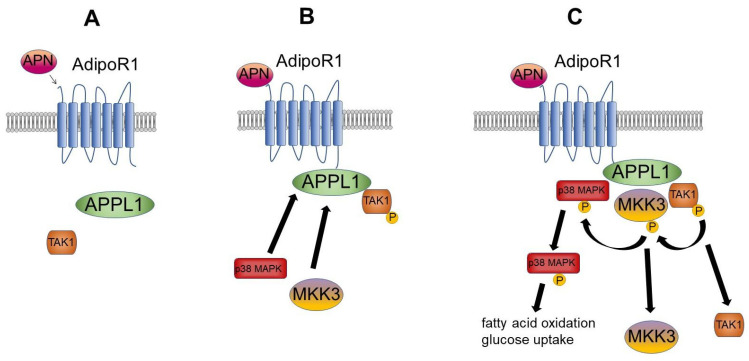
Mechanism of adiponectin-dependent MAPK activation. (**A**) In the non-stimulated state, adaptor protein containing pleckstrin homology domain, phosphotyrosine binding domain, and leucine zipper motif (APPL1) weakly interacts with transforming growth factor-β-activated kinase 1 (TAK1). (**B**) Upon adiponectin (APN) binding to the adiponectin receptor 1 (AdipoR1), APPL1 and phosphorylated TAK1 are recruited to the receptor site. This triggers the recruitment of two further proteins: p38 mitogen-activated protein kinase (p38 MAPK) and mitogen-activated protein kinase kinase 3 (MKK3). (**C**) TAK1 phosphorylates MKK3 and dissociates from the complex. MKK3 phosphorylates p38 MAPK, and both proteins dissociate from the complex. Phosphorylated p38 MAPK subsequently induces fatty acid oxidation and glucose uptake.

APPL1 also acts as a scaffold protein in p38 MAPK-mediated signaling pathways. Transforming growth factor-β-activated kinase 1 (TAK1) weakly associates with APPL1 when not bound to AdipoR (Figure 2A). Upon adiponectin signaling, APPL1 binds to AdipoR1, resulting in TAK1 phosphorylation. Upon TAK1 activation, mitogen-activated protein kinase kinase 3 (MKK3) and p38 MAPK are recruited to form the AdipoR1/APPL1/TAK1/MKK3/p38 MAPK complex (Figure 2B). After MKK3 is phosphorylated by TAK1, TAK1 dissociates from APPL1 and loses its activity. Concurrently with this, APPL1 dissociates from AdipoR1, and MKK3 phosphorylates p38 MAPK. Phosphorylated p38 MAPK dissociates from APPL1 and mediates subsequent MAPK pathway events, including increases in fatty acid oxidation and Rab5/GLUT4-mediated glucose uptake (Figure 2C) [59]. 

### 4.3. Interaction between Adiponectin and Insulin Signaling Pathways

**Figure 3:**
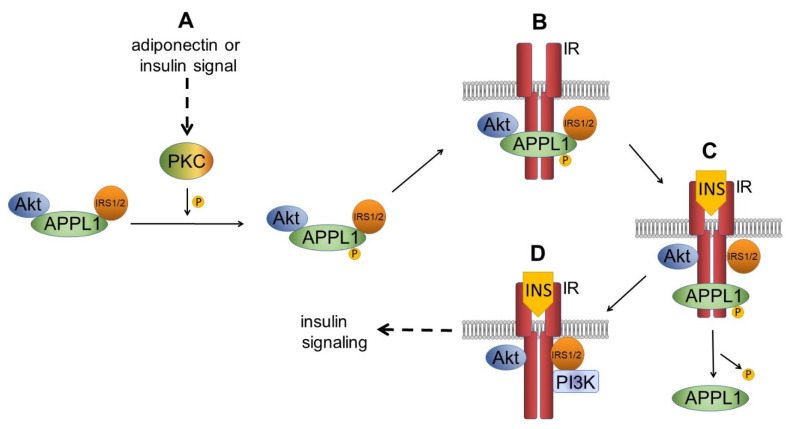
Integration of insulin and adiponectin signaling pathways. (**A**) Upon adiponectin or insulin stimulation, PKC phosphorylates APPL1. This results in insulin receptor 1/2 (IRS1/2) and Akt recruitment, resulting in APPL1/IRS1/2/Akt complex formation. This complex then associates with the insulin receptor (IR). (**B**) After the binding of insulin (INS) to the IR, APPL1 “piggybacks” Akt and IRS1/2 on the IR. (**C**) Dephosphorylated APPL1 dissociates from the complex. (**D**) IRS1/2 phosphorylates phosphatidylinositol-3 kinase (PI3K), and PI3K phosphorylates Akt, which activates its further substrates in the insulin signaling pathway.

Adiponectin also improves insulin sensitivity by inducing APPL1 to interact with insulin receptor substrate 1/2 (IRS1/2) and Akt. Under basal, non-stimulated conditions, APPL1 remains in a non-phosphorylated state in the cell and forms a complex with IRS1/2 and Akt kinase (Figure 3A). In response to adiponectin or insulin stimulation, protein kinase C (PKC) phosphorylates APPL1 at Ser407, driving the APPL1/IRS1/2/Akt complex to translocate toward the plasma membrane and bind to the insulin receptor (IR) (Figure 3A). After the binding of insulin to the IR, the APPL1/IRS1/2/Akt complex dissociates, allowing the binding of IRS1/2 to the IR, and facilitating Akt translocation to the plasma membrane (Figure 3B). After binding of insulin to IR, APPL1 is dephosphorylated, and dissociates from the IR (Figure 3C). Insulin signaling is then initiated without further interactions with APPL1 (Figure 3D) [60]. 

### 4.4. APPL1 Independent Actions of Adiponectin

Three years after APPL1 was first described, its apparent antagonist, APPL2, was identified by the same research group [61]. The interplay between APPL1 and APPL2 was termed “Yin-Yang” regulation because of their opposing effects on adiponectin signaling and cellular metabolism. APPL2 inhibits the actions of APPL1 by competing for AdipoR1 binding and hindering the APPL1-AdipoR1 interaction through APPL1 sequestration, which leads to a decrease in the phosphorylation of adiponectin intracellular messengers, such as AMPK and p38 MAPK. Additionally, APPL2 hinders adiponectin-mediated insulin-sensitizing qualities. Notably, exogenous adiponectin administration restores APPL1-mediated adiponectin signaling and increases insulin sensitivity [61]. Although APPL1 is indisputably important for adiponectin signaling, it is not crucial. Adiponectin can act in an APPL1-independent manner by increasing intracellular Ca^2+^ ion concentration by either inducing extracellular Ca^2+^ influx or increasing intracellular Ca^2+^ release. Adiponectin was shown to activate AMPK and calmodulin-dependent protein kinase kinase B (CaMKKB) by phospholipase C-mediated inositol-3-phosphate production in skeletal muscles [50]. Adiponectin and AdipoR1 signaling was also shown to be important for peroxisome proliferator-activated receptor γ coactivator-1α (PGC1α) signaling. Upon the release of Ca^2+^ ions from the endoplasmic reticulum, CaMKKB, in concert with LKB1, activates AMPK. In the next step, AMPK, together with sirtuin 1 (SIRT1) deacetylase, activates PGC1α, thereby initiating the transcription of genes that are implicated in oxidative metabolism and mitochondrial biogenesis. The adiponectin- and AdipoR1-dependent activation of CaMKKB also drives the expression of PGC1α itself by phosphorylating calmodulin-dependent protein kinase (CaMK) [36,62]. The aberrant accumulation of ceramides, which are elevated in obesity, often remain in opposition to adiponectin’s actions and sphingosine 1-phosphate (S1P) levels, which is known for its anti-apoptotic properties. S1P is converted from ceramide, glucosylceramide, and monosialodihexosylganglioside (GM3 ganglioside) [63]. Exogenous adiponectin administration using a leptin-deficient ob/ob and high-fat diet-fed mouse model of metabolic disorder was shown to promote ceramidase activity through both AdipoR1 and AdipoR2 activation, thereby improving insulin resistance and preventing caspase-8-mediated apoptosis in pancreatic β-cells, cardiomyocytes, and hepatocytes. This effect was independent of AMPK activation [64]. Recent studies proposed a mechanism by which adiponectin participates in regulating ceramidase activity. Both AdipoRs possess structural properties that suggest ceramidase activity, which can be enhanced upon adiponectin binding. Additionally, AdipoR2 has the ability to bind cellular ceramidase and enhance beneficial S1P production in cells [65]. This is consistent with a study that showed that in vitro myoblast treatment with AdipoRon (a small synthetic molecule that is known to activate both AdipoR1 and AdipoR2 [66]) enhanced ceramidase activity, resulting in higher cellular S1P levels and protecting cells against palmitate-induced lipotoxicity [67].

## 5. Effects of PVAT-Derived Adiponectin on Vascular Smooth Muscle Cell Contraction

### 5.1. Activation of AMPK Signaling

One feature that accompanies both obesity and type 2 diabetes is a chronic increase in blood pressure that leads to hypertension, mediated by constriction of the tunica media and a decrease in the secretion of vasorelaxant molecules [68,69]. Adiponectin knockout mice exhibited an increase in hypertension as a result of chronic endothelial dysfunction [70]. Therefore, PVAT was postulated to participate in adipocyte-mediated vasodilation [71]. PVAT-derived gaseous molecules, such as nitric oxide [72] and hydrogen sulfide [73], were shown to exert vasodilatory effects. Adiponectin is known to activate AMPK [36]. Some AMPK activators (e.g., AICAR), in turn, were shown to have vasodilatory properties in VSMC [74]. Adiponectin appeared to also have a vasodilatory effect [75,76,77]. AMPKα1 knockout mice exhibited no alterations of histological properties of perivascular adipose tissue or the arterial wall, but knockout mice exhibited loss of the vasodilatory effect in PVAT. Moreover, knockout mice showed markedly lower levels of circulating adiponectin, suggesting that AMPK is crucial for adiponectin secretion and adiponectin-mediated vasorelaxation [78]. Gathered data clearly show that adiponectin signaling takes part in VSMC relaxation through AMPK pathway. 

### 5.2. Large-Conductance Ca^2+^-Activated K^+^ Channels

A study used large-conductance Ca^2+^-activated K^+^ (BK_Ca_) channel knockout mice and pharmacologically inhibited BK_Ca_ channels and found that adiponectin facilitated vasodilation in pressurized mesenteric arteries, presumably by activating BK_Ca_, in which their ablation had no effect [79]. Furthermore, PVAT from BK_Ca_ knockout mice was unable to induce vasodilation in wildtype arteries, suggesting the possible involvement of these channels in adiponectin signaling. As mentioned above, three different adiponectin receptors have been identified: AdipoR1, AdipoR2, and T-cadherin [30,31]. AdipoR1 is abundantly expressed on endothelial cells and VSMCs. Adiponectin nitric oxide production has been proposed to occur via AdipoR1 in an endothelial cell-and VSMC-dependent manner [79] and play a pivotal role in acetylcholine-mediated vasodilation [80]. However, as more recent data have emerged, the direct activation of BK_Ca_ channels in VSMCs by adiponectin has been challenged. For example, Baylie et al. [81] failed to replicate the results of Lynch et al. [79]. Although pressurized arteries exhibited similar levels of vasodilation in response to adiponectin administration, patch–clamp recording in a single VSMC revealed that adiponectin administration only slightly activated BK channels, resulting in currents that were insufficient to exert a physiological response. This suggests the existence of another layer of complexity in the pathway by which PVAT-derived adiponectin acts on the vessel wall, presumably through the nervous system or other cell types, considering that only intact PVAT led to vascular relaxation [79]. 

### 5.3. Do β3-Adrenoreceptors Affect Adiponectin Signaling?

Over the years, PVAT has been considered to exert a vasorelaxant effect on vascular walls. But more recent findings refute this possibility. A study of isolated rat mesenteric arteries reported that PVAT may inhibit acetylcholine-mediated vasodilation by physically not allowing acetylcholine to penetrate through PVAT to VSMCs in the artery [82]. This physical barrier model of PVAT, however, was later questioned by Saxton et al. [83], who showed that PVAT secreted adiponectin and exerted actions on an artery that was mounted in a myograph in a paracrine manner. Furthermore, adiponectin was suggested to be secreted from PVAT as a result of β3-adrenoreceptor activation. β3-adrenoreceptor activation in PVAT was previously reported to induce nitric oxide production, and adiponectin expression remained unchanged upon β3-adrenoreceptor activation [84]. Adiponectin knockout mice exhibited higher blood pressure and alterations of glucose clearance, confirming that adiponectin is vital for blood pressure normalization in both mice and humans. The involvement of β3-adrenoreceptors in the regulation of adiponectin gene expression remains to be elucidated [83,85]. Further evidence of adiponectin-mediated vascular relaxation was provided by a recent study that confirmed previously reported results using an in vivo rat model [86].

## 6. Role of PVAT-Derived Adiponectin in Endothelial Cells

Endothelial cells comprise the tunica media, an inner layer of cells within the vessel. Therefore, the tunica media functions as a barrier between blood, the vessel, and adjacent tissues, thereby ensuring the regulation of such processes as vascular tone, immune cell recruitment, angiogenesis, blood fluidity, and blood clot formation [87]. Endothelial dysfunction is characterized by a state in which vascular endothelial cells lose their ability to induce vasodilation. 

### 6.1. Vasodilation Regulation

Adiponectin is known to facilitate vasodilation through AMPK-mediated endothelial nitric oxide synthase (eNOS) phosphorylation [88] and promote angiogenesis by inducing crosstalk between AMPK and Akt pathways [89]. The action of adiponectin on endothelial cells is ensured by both AdipoRs and T-cadherin [90,91]. Given the anatomical proximity of PVAT to the vascular endothelium, PVAT may be presumed to act as a sensor and regulator of the physiological state of endothelial cells. Indeed, among other fat tissues, PVAT was found to be the most susceptible to JNK pathway activation during the onset of type 1 diabetes, as well as adiponectin and AdipoR1 downregulation. JNK activation exerted a significant effect on endothelial cells, particularly nitric oxide production [92]. The role of exercise in the alleviation of obesity-related disorders is well established [93], the main beneficial outcomes of which arise from eNOS activity in endothelial cells [94,95]. However, PVAT eNOS activity was only recently shown to have a significant impact on endothelial function as well. In rats that were fed a high-fat/high-sucrose diet and were subjected to exercise, adiponectin levels significantly increased compared with non-exercised control rats. Interestingly, this increase was associated with an increase in eNOS phosphorylation in both PVAT and endothelial cells, which led the authors to conclude that adiponectin-mediated vasodilation originates in both periadventitial adipocytes and the vascular endothelium [96]. PVAT may also act as a backup source of nitric oxide in the case of endothelial dysfunction. PVAT-derived adiponectin might be involved in this compensatory effect. Obese patients with a body mass index of 25–30 kg/m^2^ presented higher adiponectin PVAT expression compared with lean patients [97]. Hypercholesterolemic low-density lipoprotein receptor (LDLr) knockout mice exhibited the rescue of vascular relaxation in thoracic aortas with intact PVAT compared with PVAT-denuded aortas. This effect, however, was not associated with alterations of adiponectin expression [98]. This effect may be related to adipocyte β3-adrenoreceptor activation, which was shown to mediate vasorelaxation-induced norepinephrine release [84]. Gestational intermittent hypoxia that was caused by obstructive sleep apnea was shown to influence PVAT-mediated endothelial dysfunction. Male offspring of women who experienced obstructive sleep apnea exhibited impairments in endothelium-dependent vasodilation, which also correlated with a higher susceptibility to insulin resistance [99]. PVAT-mediated vasodilation was also absent in male offspring of female mice that were subjected to hypoxia during pregnancy and in male offspring of female rats that were fed a high-fat diet before and during pregnancy. In both cases, loss of the vasorelaxant effect of PVAT was associated with lower adiponectin content in PVAT. These findings suggest that adiponectin secretion can decrease when PVAT is exposed to hypoxic conditions. Intriguingly, this effect is heritable, presumably through epigenetic modifications that may include adiponectin gene promoter hypermethylation [100,101]. Another interesting example of the complex regulation of vascular tone that is mediated by the adipovascular axis is early life stress. A previous study using a rat model of maternal separation, showed that PVAT adiponectin induced relaxation in the early life stress group that was subjected to a high-fat diet compared with the non-stressed control group [102]. This finding may be related to the fact that although adiponectin is downregulated in obese adipose tissue, its mRNA and protein expression can be detected in the tunica intima, suggesting an adipose tissue-independent source of this adipokine [103]. Considering the immediate proximity of PVAT and endothelial cells, several attempts have been made to therapeutically induce the adiponectin-dependent improvement of endothelial dysfunction. For example, methotrexate was shown to induce activation of the AMPK/eNOS pathway in both PVAT and endothelial cells [104]. Calycosin is a major bioactive isoflavonoid with known anti-inflammatory and antioxidative properties. Calycosin treatment reversed obesity-induced PVAT inflammation and improved adiponectin-dependent vasodilation [105]. A group of flavonoids, termed anthocyanins, that are present in a large number of colorful fruits, red wine, and vegetables was shown to improve insulin resistance and lower the risk of type 2 diabetes [106,107] and cardiovascular disease [108]. Anthocyanin cyanidin-3-O-β-glucoside (C3G) is another molecule with a proven ability to improve endothelial function through adiponectin in diabetic mice via the SIRT1/forkhead box protein O1 (FOXO1) pathway [109]. C3G also exerted a beneficial effect on the endothelium in atherogenic LDLr knockout mice [110]. Despite the need for further studies to fully elucidate the aforementioned phenomena, adiponectin signaling in PVAT has clearly emerged as a potential therapeutic target for cardiovascular and metabolic disorders.

### 6.2. Angiogenesis Process

Adipose tissue exhibits robust volume plasticity. Its ability to change its volume during life is an exceptional property among other tissues. This flexibility, however, must be orchestrated with sufficient blood vessel formation during expansion and involution in the case of adipose tissue shrinkage. Inadequate angiogenesis during adipose tissue development results in hypoxia, which is a hallmark of obesity [111]. A major controller of angiogenesis is the vascular endothelial growth factor (VEGF). Mice that overexpressed VEGF exhibited an increase in adipose tissue vascularization and were protected against obesity-induced metabolic syndrome [112,113,114]. Consistent with these findings, VEGF ablation resulted in an increase in WAT inflammation, BAT lipid accumulation, and impairments in insulin sensitivity [115,116]. Recent studies also showed that VEGF-A ablation exclusively in adipose tissue had a negative impact on PVAT vascularization and collagen deposition within adipose tissue, suggesting its fibrosis. Moreover, VEGF ablation decreased UCP1 expression, suggesting that the BAT phenotype of thoracic PVAT is maintained by VEGF. Importantly, the aforementioned alterations were associated with adiponectin downregulation and a decrease in aortic relaxation, implying that PVAT micro vascularization may be responsible for macrovascular responsiveness through adiponectin signaling [117].

## 7. Role of PVAT-Derived Adiponectin in Atherosclerosis

Atherosclerosis development is a multifactorial process that occurs in blood vessels, resulting in narrowing of the arterial lumen that can lead to severe ischemia. This is particularly interesting because when atherosclerosis occurs in coronary arteries, it can cause myocardial infarction. Atherosclerotic plaque formation is initiated when endothelial cells in the arterial tunica intima begin to capture passing leukocytes and allow them to penetrate the extracellular matrix. Extracellular matrix invasion by leukocytes is facilitated by cell–cell junction relaxation between endothelial cells, which increases endothelial cell permeability. This then results in cholesterol deposition that is bound to LDL within the extracellular matrix. Recruited monocytes, which are most abundant within nascent plaques, differentiate into macrophages and engulf LDL deposits, forming lipid-laden mononuclear phagocytes called foam cells. In the next stage, smooth muscle cells from the tunica media migrate toward newly forming plaques and change their phenotype from contractile to synthetic. This phenotypic switch has been described as an increase in extracellular matrix protein expression and secretion, which leads to atherosclerotic lesion formation. The physical disruption of atherosclerotic lesion triggers blood clot formation, which can later detach from the vascular wall and block blood flow in narrower sections of the vessel [118].

Adiponectin is known for its antiatherosclerotic properties [119], consisting of its ability to inhibit the proinflammatory, classic activation of macrophages (i.e., M1 macrophages) and promote their alternative, anti-inflammatory activation (i.e., M2 macrophages) [120,121], prevent macrophage-to-foam cell formation [122,123,124,125], impede VSMC proliferation and migration [126], and inhibit endothelial cell activation upon proinflammatory stimulation, such as by tumor necrosis factor α (TNFα), in a nuclear factor kappa-light-chain-enhancer of activated B cells (NF-κB)-dependent manner [127,128]. Occlusion of the arterial lumen in atherosclerosis often occurs when growing plaques detach from the disease-altered vessel and travel with blood. Within atherosclerotic lesions, immobilized LDL undergoes oxidation as a result of reactive oxygen species biogenesis. This leads to oxLDL formation, which in turn induces matrix metalloproteinase-9 (MMP-9) activity. MMP-9 plays an important role in plaque rupture. Adiponectin was reported to inhibit this process in VSMCs, partially by maintaining proper antioxidant levels (e.g., glutathione) and partially by inhibiting MMP-9 activity [129,130]. Most studies of adiponectin signaling in atherosclerosis have focused on systemic adiponectin or adiponectin signaling itself [131]. Accumulating evidence, however, suggests that the origin of adiponectin is also important in this process. Autophagy is considered beneficial in terms of defending against atherosclerosis because of its ability to induce intracellular debris degradation and promote cell survival [132]. Autophagy has mainly been studied in VSMCs and endothelial cells [133,134]. PVAT-derived adiponectin was reported to have antiatherogenic properties because of its ability to induce Akt/FOXO3 dependent autophagy in macrophages in a model of collar-induced carotid atherosclerosis [135]. In humans, a special PVAT depot that surrounds coronary vasculature is termed epicardial adipose tissue. Epicardial adipose tissue in individuals who suffer from coronary artery disease exhibits an increase in proinflammatory cytokines, such as leptin and interleukin-6, with a simultaneous decrease in the epicardial adipose tissue-specific production of adiponectin. This emphasizes the fact that low adiponectin levels are a marker of poor prognosis in cardiovascular disease [136]. Vitamin A is a well-established regulator of lipid metabolism, inflammation, and many other factors that contribute to cardiovascular disease [137]. Among a plethora of different vitamin A metabolites, two appear to have particular clinical importance: 9-cis retinoic acid and all-trans-retinoic acid (ATRA) [138]. Among these two, ATRA was shown to exert protective effects against cardiovascular conditions through anti-inflammatory and antioxidant actions [139,140]. ATRA treatment was shown to significantly increase adiponectin secretion in PVAT using an apolipoprotein E knockout mouse model of atherosclerosis. Interestingly, this effect was not observed in visceral adipose tissue, thus underscoring the importance of local adipose tissue depots in cardiovascular disease [141].

## 8. Adiponectin Signaling as a Potential Therapeutic Target

Emerging evidence suggests that adiponectin that is secreted into the circulation by remote fat pads is clinically important in terms of vascular biology. Local adiponectin secretion from PVAT appears to act on vascular wall layers in presumably a paracrine fashion or through the network of small blood vessels supplying walls of large blood vessels (vasa vasorum). 

### 8.1. Strategies for Increasing Circulating Adiponectin

Most research efforts to test adiponectin as a therapeutic target have focused on increasing its plasma levels [142]. Pharmacological manipulations to increase plasma adiponectin levels have included thiazolidinediones, such as rosiglitazone, which are PPARγ agonists [143,144]. The insulin-sensitizing properties of thiazolidinediones are reported to be at least partially mediated by adiponectin [145]. Despite promising outcomes of thiazolidinedione treatment, some safety concerns have been raised. Prolonged thiazolidinedione treatment was associated with a higher risk of heart failure [146], edema development in type 2 diabetic patients [147], and lower bone density, and a higher risk of bone fracture [148]. Because of these negative outcomes, other agents have been tested that can selectively activate PPARγ. For example, INT131 is a non-thiazolidinedione PPARγ modulator that has significantly fewer side effects with preserved insulin-sensitizing properties [149]. Moreover, selective targeting of the renin–angiotensin–aldosterone axis increased adiponectin levels in obese Zucker rats [150]. The pharmacological blockade of angiotensin receptors was shown to activate PPARγ in humans [151]. Some plant-derived agents that can increase adiponectin have also been identified. In rats that were fed a high-fructose diet, Zataria multiflora extract treatment increased adiponectin levels [152]. Similar effects were found in a human study, in which garlic extract increased adiponectin levels in patients with metabolic syndrome [153]. Aerobic exercise is another therapeutic strategy that has well-established beneficial outcomes. In male subjects, adiponectin levels significantly increased after sculling exercise [154,155], suggesting a promising way to increase adiponectin levels without the need for pharmacological interventions. Physiological levels of adiponectin are relatively high, and its production is diminished in obesity [23,156]. Clinical trials have tested exogenous adiponectin administration to improve metabolic outcomes. However, the half-life of this adipokine is relatively short (~75 min) [157], which makes it unappealing for administration. Furthermore, despite correct folding and posttranslational modifications, recombinant adiponectin failed to impact glucose levels in diabetic db/db and ob/ob mice [158].

### 8.2. Adiponectin Paradox

When considering adiponectin as a potential therapeutic target, it is worth mentioning the so-called ‘adiponectin paradox’ phenomenon which is described by high plasma concentrations of adiponectin in patients suffering from severe cardiovascular dysfunction [156,159]. Given adiponectin’s anti-inflammatory and antiatherosclerotic properties, higher concentrations of this adipokine should be inversely correlated with the prevalence of cardiovascular events [159]. However, in some cases, higher adiponectin levels show a correlation with increased cardiovascular risk, especially in patients with other metabolic conditions, e.g., type 2 diabetes [160,161,162]. This phenomenon can be at least partly explained by impaired liver function in studied subjects since adiponectin is degraded in the liver [157,159]. It seems plausible that higher adiponectin levels in those patients are a result of impaired liver function and are a secondary feature of metabolic disorders rather than a primary cause of cardiovascular complications. Another possible explanation for the adiponectin paradox is that high molecular weight (HMW) adiponectin is the most active form, with most cardioprotective properties, and available studies mostly focus on total adiponectin levels rather than its quality [159]. However, it has been shown that HMW adiponectin is not a good predictor of chronic heart failure, indicating that the form of adiponectin in circulation may play a vital role in cardiovascular disorders rather than only its plasma concentrations [163,164]. More studies, possibly utilizing molecular and genetic approaches, are required to fully address this issue since presented data only show associations without any mechanistic insights.

### 8.3. Pharmacological Activation of AdipoRs

AdipoRon, a small-molecule dual agonist of both AdipoR1 and AdipoR2, was developed by Okada-Iwabu et al. [66]. AdipoRon exerted actions on muscle and liver cells by activating the PPARα and AMPK pathways and ameliorated insulin resistance in diabetic mice [66]. Since the discovery of AdipoRon, it has been shown to exert various effects in such conditions as diabetic nephropathy [165], post-ischemia-reperfusion heart failure [166], depression [167], and cancer [168,169]. AdipoRon also has actions on vascular relaxation through a direct action on VSMCs. It was demonstrated to still be effective in endothelium-denuded arteries. Its actions were shown to be mediated by BK_Ca_ channel activation rather than through AMPK, in which compound C did not inhibit the observed phenomenon. Intriguingly, AdipoRon appears to be an even more potent vasodilator than adiponectin itself [170].

### 8.4. Adiponectin in Aging

Most efforts concerning adiponectin signaling in murine models were focused on metabolic outcomes of adiponectin in relatively young animals (around 20 weeks). A recent study conducted by Li et al. provided an insight into the potential role of adiponectin in the longevity of mice older than 100 weeks [171]. It has been demonstrated, using both adiponectin knockout mice and mice overexpressing adiponectin, that lack of adiponectin induces fibrosis in multiple organs, including kidneys and liver, and this effect is mediated partly by an increased inflammatory response in knockout mice. This effect is reversed in mice overexpressing adiponectin. Moreover, mice overexpressing adiponectin showed an increased lifespan by 9%, and adiponectin knockout led to shortened lifespan and acceleration of age-related disorders occurrence. These findings are supported by previous human research examining the effects of TZDs on age-related tissue damage [172,173].

## 9. Conclusions

In summary, we presented that adiponectin is involved in many physiological processes and that it plays beneficial roles in maintaining vascular homeostasis. The vast majority of studies concerning adiponectin signaling are focused on the endocrine function of this adipokine. Here, we emphasized the evidence that paracrine actions of adiponectin, especially in PVAT, must not be omitted when considering adiponectin’s therapeutic applications. Possibly, future experiments should be more focused on local adiponectin signaling activation, which would allow for the development of selective adiponectin activators. Another interesting approach, presented by Zhao et al. [159], implies that novel therapeutics should encompass not only adiponectin signaling but also other adipokines, e.g., leptin, which are also dysregulated in obesity and CVD. Such findings could lead to the discovery of therapeutic strategies allowing for more efficient treatment of such conditions as aneurysms, atherosclerosis, or hypertension.

## Data Availability

Not applicable.

## References

[1] Saxton S.N., Clark B.J., Withers S.B., Eringa E.C., Heagerty A.M. (2019). Mechanistic Links between Obesity, Diabetes, and Blood Pressure: Role of Perivascular Adipose Tissue. Physiol. Rev..

[2] Trayhurn P., Wood I.S. (2004). Adipokines: Inflammation and the pleiotropic role of white adipose tissue. Br. J. Nutr..

[3] Trayhurn P., Wood I.S. (2005). Signalling role of adipose tissue: Adipokines and inflammation in obesity. Biochem. Soc. Trans..

[4] Qi X.Y., Qu S.L., Xiong W.H., Rom O., Chang L., Jiang Z.S. (2018). Perivascular adipose tissue (PVAT) in atherosclerosis: A double-edged sword. Cardiovasc. Diabetol..

[5] Scherer P.E., Williams S., Fogliano M., Baldini G., Lodish H.F. (1995). A novel serum protein similar to C1q, produced exclusively in adipocytes. J. Biol. Chem..

[6] Yamauchi T., Kamon J., Minokoshi Y., Ito Y., Waki H., Uchida S., Yamashita S., Noda M., Kita S., Ueki K. (2002). Adiponectin stimulates glucose utilization and fatty-acid oxidation by activating AMP-activated protein kinase. Nat. Med..

[7] Ceddia R.B., Somwar R., Maida A., Fang X., Bikopoulos G., Sweeney G. (2005). Globular adiponectin increases GLUT4 translocation and glucose uptake but reduces glycogen synthesis in rat skeletal muscle cells. Diabetologia.

[8] Yoon M.J., Lee G.Y., Chung J.J., Ahn Y.H., Hong S.H., Kim J.B. (2006). Adiponectin increases fatty acid oxidation in skeletal muscle cells by sequential activation of AMP-activated protein kinase, p38 mitogen-activated protein kinase, and peroxisome proliferator-activated receptor alpha. Diabetes.

[9] Wang C., Mao X., Wang L., Liu M., Wetzel M.D., Guan K.L., Dong L.Q., Liu F. (2007). Adiponectin sensitizes insulin signaling by reducing p70 S6 kinase-mediated serine phosphorylation of IRS-1. J. Biol. Chem..

[10] Ouchi N., Walsh K. (2007). Adiponectin as an anti-inflammatory factor. Clin. Chim Acta..

[11] Soltis E.E., Cassis L.A. (1991). Influence of perivascular adipose tissue on rat aortic smooth muscle responsiveness. Clin. Exp. Hypertens. A.

[12] Löhn M., Dubrovska G., Lauterbach B., Luft F.C., Gollasch M., Sharma A.M. (2002). Periadventitial fat releases a vascular relaxing factor. FASEB J..

[13] Dubrovska G., Verlohren S., Luft F.C., Gollasch M. (2004). Mechanisms of ADRF release from rat aortic adventitial adipose tissue. Am. J. Physiol Heart Circ. Physiol..

[14] Rosen E.D., Spiegelman B.M. (2014). What we talk about when we talk about fat. Cell.

[15] Fitzgibbons T.P., Kogan S., Aouadi M., Hendricks G.M., Straubhaar J., Czech M.P. (2011). Similarity of mouse perivascular and brown adipose tissues and their resistance to diet-induced inflammation. Am. J. Physiol Heart Circ. Physiol..

[16] Padilla J., Jenkins N.T., Vieira-Potter V.J., Laughlin M.H. (2013). Divergent phenotype of rat thoracic and abdominal perivascular adipose tissues. Am. J. Physiol. Regul. Integr. Comp. Physiol..

[17] Brown N.K., Zhou Z., Zhang J., Zeng R., Wu J., Eitzman D.T., Chen Y.E., Chang L. (2014). Perivascular adipose tissue in vascular function and disease: A review of current research and animal models. Arterioscler. Thromb. Vasc. Biol..

[18] Horimatsu T., Kim H.W., Weintraub N.L. (2017). The Role of Perivascular Adipose Tissue in Non-atherosclerotic Vascular Disease. Front. Physiol..

[19] Piacentini L., Werba J.P., Bono E., Saccu C., Tremoli E., Spirito R., Colombo G.I. (2019). Genome-Wide Expression Profiling Unveils Autoimmune Response Signatures in the Perivascular Adipose Tissue of Abdominal Aortic Aneurysm. Arterioscler. Thromb. Vasc. Biol..

[20] Villacorta L., Chang L. (2015). The role of perivascular adipose tissue in vasoconstriction, arterial stiffness, and aneurysm. Horm. Mol. Biol. Clin. Investig..

[21] Nakano Y., Tobe T., Choi-Miura N.H., Mazda T., Tomita M. (1996). Isolation and characterization of GBP28, a novel gelatin-binding protein purified from human plasma. J. Biochem..

[22] Das K., Lin Y., Widen E., Zhang Y., Scherer P.E. (2001). Chromosomal localization, expression pattern, and promoter analysis of the mouse gene encoding adipocyte-specific secretory protein Acrp30. Biochem. Biophys. Res. Commun..

[23] Pajvani U.B., Du X., Combs T.P., Berg A.H., Rajala M.W., Schulthess T., Engel J., Brownlee M., Scherer P.E. (2003). Structure-function studies of the adipocyte-secreted hormone Acrp30/adiponectin. Implications for metabolic regulation and bioactivity. J. Biol. Chem..

[24] Magkos F., Sidossis L.S. (2007). Recent advances in the measurement of adiponectin isoform distribution. Curr. Opin. Clin. Nutr. Metab. Care.

[25] Fruebis J., Tsao T.S., Javorschi S., Ebbets-Reed D., Erickson M.R., Yen F.T., Bihain B.E., Lodish H.F. (2001). Proteolytic cleavage product of 30-kDa adipocyte complement-related protein increases fatty acid oxidation in muscle and causes weight loss in mice. Proc. Natl. Acad. Sci. USA.

[26] Hotta K., Funahashi T., Arita Y., Takahashi M., Matsuda M., Okamoto Y., Iwahashi H., Kuriyama H., Ouchi N., Maeda K. (2000). Plasma concentrations of a novel, adipose-specific protein, adiponectin, in type 2 diabetic patients. Arterioscler. Thromb. Vasc. Biol..

[27] Maeda N., Shimomura I., Kishida K., Nishizawa H., Matsuda M., Nagaretani H., Furuyama N., Kondo H., Takahashi M., Arita Y. (2002). Diet-induced insulin resistance in mice lacking adiponectin/ACRP30. Nat. Med..

[28] Kubota N., Terauchi Y., Yamauchi T., Kubota T., Moroi M., Matsui J., Eto K., Yamashita T., Kamon J., Satoh H. (2002). Disruption of adiponectin causes insulin resistance and neointimal formation. J. Biol. Chem..

[29] Kakino A., Fujita Y., Ke L.Y., Chan H.C., Tsai M.H., Dai C.Y., Chen C.H., Sawamura T. (2020). Adiponectin forms a complex with atherogenic LDL and inhibits its downstream effects. J. Lipid Res..

[30] Yamauchi T., Kamon J., Ito Y., Tsuchida A., Yokomizo T., Kita S., Sugiyama T., Miyagishi M., Hara K., Tsunoda M. (2003). Cloning of adiponectin receptors that mediate antidiabetic metabolic effects. Nature.

[31] Hug C., Wang J., Ahmad N.S., Bogan J.S., Tsao T.S., Lodish H.F. (2004). T-cadherin is a receptor for hexameric and high-molecular-weight forms of Acrp30/adiponectin. Proc. Natl. Acad. Sci. USA.

[32] Yokomizo T., Izumi T., Chang K., Takuwa Y., Shimizu T. (1997). A G-protein-coupled receptor for leukotriene B4 that mediates chemotaxis. Nature.

[33] Scheer A., Fanelli F., Costa T., de Benedetti P.G., Cotecchia S. (1996). Constitutively active mutants of the alpha 1B-adrenergic receptor: Role of highly conserved polar amino acids in receptor activation. EMBO J..

[34] Wess J. (1997). G-protein-coupled receptors: Molecular mechanisms involved in receptor activation and selectivity of G-protein recognition. FASEB J..

[35] Yamauchi T., Nio Y., Maki T., Kobayashi M., Takazawa T., Iwabu M., Okada-Iwabu M., Kawamoto S., Kubota N., Kubota T. (2007). Targeted disruption of AdipoR1 and AdipoR2 causes abrogation of adiponectin binding and metabolic actions. Nat. Med..

[36] Iwabu M., Yamauchi T., Okada-Iwabu M., Sato K., Nakagawa T., Funata M., Yamaguchi M., Namiki S., Nakayama R., Tabata M. (2010). Adiponectin and AdipoR1 regulate PGC-1alpha and mitochondria by Ca(2+) and AMPK/SIRT1. Nature.

[37] Bjursell M., Ahnmark A., Bohlooly Y.M., William-Olsson L., Rhedin M., Peng X.R., Ploj K., Gerdin A.K., Arnerup G., Elmgren A. (2007). Opposing effects of adiponectin receptors 1 and 2 on energy metabolism. Diabetes.

[38] Liu Y., Michael M.D., Kash S., Bensch W.R., Monia B.P., Murray S.F., Otto K.A., Syed S.K., Bhanot S., Sloop K.W. (2007). Deficiency of adiponectin receptor 2 reduces diet-induced insulin resistance but promotes type 2 diabetes. Endocrinology.

[39] Lyons T.J., Villa N.Y., Regalla L.M., Kupchak B.R., Vagstad A., Eide D.J. (2004). Metalloregulation of yeast membrane steroid receptor homologs. Proc. Natl. Acad. Sci. USA.

[40] Kupchak B.R., Garitaonandia I., Villa N.Y., Mullen M.B., Weaver M.G., Regalla L.M., Kendall E.A., Lyons T.J. (2007). Probing the mechanism of FET3 repression by Izh2p overexpression. Biochim. Biophys. Acta..

[41] Devkota R., Svensk E., Ruiz M., Ståhlman M., Borén J., Pilon M. (2017). The adiponectin receptor AdipoR2 and its Caenorhabditis elegans homolog PAQR-2 prevent membrane rigidification by exogenous saturated fatty acids. PLoS Genet..

[42] Pilon M. (2021). Paradigm shift: The primary function of the “Adiponectin Receptors” is to regulate cell membrane composition. Lipids Health Dis..

[43] Ruiz M., Ståhlman M., Borén J., Pilon M. (2019). AdipoR1 and AdipoR2 maintain membrane fluidity in most human cell types and independently of adiponectin. J. Lipid Res..

[44] Ruiz M., Palmgren H., Henricsson M., Devkota R., Jaiswal H., Maresca M., Bohlooly-Y M., Peng X.R., Borén J., Pilon M. (2021). Extensive transcription mis-regulation and membrane defects in AdipoR2-deficient cells challenged with saturated fatty acids. Biochim. Biophys. Acta Mol. Cell Biol. Lipids.

[45] Ranscht B., Dours-Zimmermann M.T. (1991). T-cadherin, a novel cadherin cell adhesion molecule in the nervous system lacks the conserved cytoplasmic region. Neuron.

[46] Fujita K., Maeda N., Sonoda M., Ohashi K., Hibuse T., Nishizawa H., Nishida M., Hiuge A., Kurata A., Kihara S. (2008). Adiponectin protects against angiotensin II-induced cardiac fibrosis through activation of PPAR-alpha. Arterioscler. Thromb. Vasc. Biol..

[47] Parker-Duffen J.L., Nakamura K., Silver M., Kikuchi R., Tigges U., Yoshida S., Denzel M.S., Ranscht B., Walsh K. (2013). T-cadherin is essential for adiponectin-mediated revascularization. J. Biol. Chem..

[48] Fujishima Y., Maeda N., Matsuda K., Masuda S., Mori T., Fukuda S., Sekimoto R., Yamaoka M., Obata Y., Kita S. (2017). Adiponectin association with T-cadherin protects against neointima proliferation and atherosclerosis. FASEB J..

[49] Tanaka Y., Kita S., Nishizawa H., Fukuda S., Fujishima Y., Obata Y., Nagao H., Masuda S., Nakamura Y., Shimizu Y. (2019). Adiponectin promotes muscle regeneration through binding to T-cadherin. Sci. Rep..

[50] Matsuda K., Fujishima Y., Maeda N., Mori T., Hirata A., Sekimoto R., Tsushima Y., Masuda S., Yamaoka M., Inoue K. (2015). Positive feedback regulation between adiponectin and T-cadherin impacts adiponectin levels in tissue and plasma of male mice. Endocrinology.

[51] Chung C.M., Lin T.H., Chen J.W., Leu H.B., Yang H.C., Ho H.Y., Ting C.T., Sheu S.H., Tsai W.C., Chen J.H. (2011). A genome-wide association study reveals a quantitative trait locus of adiponectin on CDH13 that predicts cardiometabolic outcomes. Diabetes.

[52] Morisaki H., Yamanaka I., Iwai N., Miyamoto Y., Kokubo Y., Okamura T., Okayama A., Morisaki T. (2012). CDH13 gene coding T-cadherin influences variations in plasma adiponectin levels in the Japanese population. Hum. Mutat..

[53] Gao H., Kim Y.M., Chen P., Igase M., Kawamoto R., Kim M.K., Kohara K., Lee J., Miki T., Ong R.T. (2013). Genetic variation in CDH13 is associated with lower plasma adiponectin levels but greater adiponectin sensitivity in East Asian populations. Diabetes.

[54] Obata Y., Kita S., Koyama Y., Fukuda S., Takeda H., Takahashi M., Fujishima Y., Nagao H., Masuda S., Tanaka Y. (2018). Adiponectin/T-cadherin system enhances exosome biogenesis and decreases cellular ceramides by exosomal release. JCI Insight.

[55] Kita S., Fukuda S., Maeda N., Shimomura I. (2019). Native adiponectin in serum binds to mammalian cells expressing T-cadherin, but not AdipoRs or calreticulin. Elife.

[56] Mao X., Kikani C.K., Riojas R.A., Langlais P., Wang L., Ramos F.J., Fang Q., Christ-Roberts C.Y., Hong J.Y., Kim R.Y. (2006). APPL1 binds to adiponectin receptors and mediates adiponectin signalling and function. Nat. Cell Biol..

[57] Zhou L., Deepa S.S., Etzler J.C., Ryu J., Mao X., Fang Q., Liu D.D., Torres J.M., Jia W., Lechleiter J.D. (2009). Adiponectin activates AMP-activated protein kinase in muscle cells via APPL1/LKB1-dependent and phospholipase C/Ca^2+^/Ca^2+^/calmodulin-dependent protein kinase kinase-dependent pathways. J. Biol. Chem..

[58] Deepa S.S., Zhou L., Ryu J., Wang C., Mao X., Li C., Zhang N., Musi N., DeFronzo R.A., Liu F. (2011). APPL1 mediates adiponectin-induced LKB1 cytosolic localization through the PP2A-PKCzeta signaling pathway. Mol. Endocrinol..

[59] Xin X., Zhou L., Reyes C.M., Liu F., Dong L.Q. (2011). APPL1 mediates adiponectin-stimulated p38 MAPK activation by scaffolding the TAK1-MKK3-p38 MAPK pathway. Am. J. Physiol. Endocrinol. Metab..

[60] Ryu J., Galan A.K., Xin X., Dong F., Abdul-Ghani M.A., Zhou L., Wang C., Li C., Holmes B.M., Sloane L.B. (2014). APPL1 potentiates insulin sensitivity by facilitating the binding of IRS1/2 to the insulin receptor. Cell Rep..

[61] Wang C., Xin X., Xiang R., Ramos F.J., Liu M., Lee H.J., Chen H., Mao X., Kikani C.K., Liu F. (2009). Yin-Yang regulation of adiponectin signaling by APPL isoforms in muscle cells. J. Biol. Chem..

[62] Handschin C., Spiegelman B.M. (2008). The role of exercise and PGC1alpha in inflammation and chronic disease. Nature.

[63] Takabe K., Paugh S.W., Milstien S., Spiegel S. (2008). “Inside-out” signaling of sphingosine-1-phosphate: Therapeutic targets. Pharmacol. Rev..

[64] Holland W.L., Miller R.A., Wang Z.V., Sun K., Barth B.M., Bui H.H., Davis K.E., Bikman B.T., Halberg N., Rutkowski J.M. (2011). Receptor-mediated activation of ceramidase activity initiates the pleiotropic actions of adiponectin. Nat. Med..

[65] Vasiliauskaité-Brooks I., Sounier R., Rochaix P., Bellot G., Fortier M., Hoh F., de Colibus L., Bechara C., Saied E.M., Arenz C. (2017). Structural insights into adiponectin receptors suggest ceramidase activity. Nature.

[66] Okada-Iwabu M., Yamauchi T., Iwabu M., Honma T., Hamagami K., Matsuda K., Yamaguchi M., Tanabe H., Kimura-Someya T., Shirouzu M. (2013). A small-molecule AdipoR agonist for type 2 diabetes and short life in obesity. Nature.

[67] Botta A., Elizbaryan K., Tashakorinia P., Lam N.H., Sweeney G. (2020). An adiponectin-S1P autocrine axis protects skeletal muscle cells from palmitate-induced cell death. Lipids Health Dis..

[68] Schulz E., Gori T., Münzel T. (2011). Oxidative stress and endothelial dysfunction in hypertension. Hypertens. Res..

[69] London G.M., Cohn J.N. (2002). Prognostic application of arterial stiffness: Task forces. Am. J. Hypertens..

[70] Cao Y., Tao L., Yuan Y., Jiao X., Lau W.B., Wang Y., Christopher T., Lopez B., Chan L., Goldstein B. (2009). Endothelial dysfunction in adiponectin deficiency and its mechanisms involved. J. Mol. Cell Cardiol..

[71] Lee R.M., Lu C., Su L.Y., Werstuck G., Gao Y.J. (2009). Effects of hyperglycemia on the modulation of vascular function by perivascular adipose tissue. J. Hypertens..

[72] Gao Y.J. (2007). Dual modulation of vascular function by perivascular adipose tissue and its potential correlation with adiposity/lipoatrophy-related vascular dysfunction. Curr. Pharm. Des..

[73] Fang L., Zhao J., Chen Y., Ma T., Xu G., Tang C., Liu X., Geng B. (2009). Hydrogen sulfide derived from periadventitial adipose tissue is a vasodilator. J. Hypertens..

[74] Rubin L.J., Magliola L., Feng X., Jones A.W., Hale C.C. (1985). Metabolic activation of AMP kinase in vascular smooth muscle. J. Appl. Physiol..

[75] Fésüs G., Dubrovska G., Gorzelniak K., Kluge R., Huang Y., Luft F.C., Gollasch M. (2007). Adiponectin is a novel humoral vasodilator. Cardiovasc. Res..

[76] Greenstein A.S., Khavandi K., Withers S.B., Sonoyama K., Clancy O., Jeziorska M., Laing I., Yates A.P., Pemberton P.W., Malik R.A. (2009). Local inflammation and hypoxia abolish the protective anticontractile properties of perivascular fat in obese patients. Circulation.

[77] Meijer R.I., Bakker W., Alta C.L., Sipkema P., Yudkin J.S., Viollet B., Richter E.A., Smulders Y.M., van Hinsbergh V.W., Serné E.H. (2013). Perivascular adipose tissue control of insulin-induced vasoreactivity in muscle is impaired in db/db mice. Diabetes.

[78] Almabrouk T., Ugusman A.B., Katwan O.J., Salt I.P., Kennedy S. (2017). Deletion of AMPKα1 attenuates the anticontractile effect of perivascular adipose tissue (PVAT) and reduces adiponectin release. Br. J. Pharmacol..

[79] Lynch F.M., Withers S.B., Yao Z., Werner M.E., Edwards G., Weston A.H., Heagerty A.M. (2013). Perivascular adipose tissue-derived adiponectin activates BK(Ca) channels to induce anticontractile responses. Am. J. Physiol. Heart Circ. Physiol..

[80] Du Y., Li R., Lau W.B., Zhao J., Lopez B., Christopher T.A., Ma X.L., Wang Y. (2016). Adiponectin at Physiologically Relevant Concentrations Enhances the Vasorelaxative Effect of Acetylcholine via Cav-1/AdipoR-1 Signaling. PLoS ONE.

[81] Baylie R., Ahmed M., Bonev A.D., Hill-Eubanks D.C., Heppner T.J., Nelson M.T., Greenstein A.S. (2017). Lack of direct effect of adiponectin on vascular smooth muscle cell BKCa channels or Ca^2+^ signaling in the regulation of small artery pressure-induced constriction. Physiol. Rep..

[82] Li R., Andersen I., Aleke J., Golubinskaya V., Gustafsson H., Nilsson H. (2013). Reduced anti-contractile effect of perivascular adipose tissue on mesenteric small arteries from spontaneously hypertensive rats: Role of Kv7 channels. Eur. J. Pharmacol..

[83] Saxton S.N., Ryding K.E., Aldous R.G., Withers S.B., Ohanian J., Heagerty A.M. (2018). Role of Sympathetic Nerves and Adipocyte Catecholamine Uptake in the Vasorelaxant Function of Perivascular Adipose Tissue. Arterioscler. Thromb. Vasc. Biol..

[84] Bussey C.E., Withers S.B., Saxton S.N., Bodagh N., Aldous R.G., Heagerty A.M. (2018). β3 -Adrenoceptor stimulation of perivascular adipocytes leads to increased fat cell-derived NO and vascular relaxation in small arteries. Br. J. Pharmacol..

[85] Aghamohammadzadeh R., Greenstein A.S., Yadav R., Jeziorska M., Hama S., Soltani F., Pemberton P.W., Ammori B., Malik R.A., Soran H. (2013). Effects of bariatric surgery on human small artery function: Evidence for reduction in perivascular adipocyte inflammation, and the restoration of normal anticontractile activity despite persistent obesity. J. Am. Coll. Cardiol..

[86] Saxton S.N., Withers S.B., Nyvad J., Mazur A., Matchkov V., Heagerty A.M., Aalkjær C. (2019). Perivascular Adipose Tissue Contributes to the Modulation of Vascular Tone in vivo. J. Vasc. Res..

[87] Krüger-Genge A., Blocki A., Franke R.P., Jung F. (2019). Vascular Endothelial Cell Biology: An Update. Int. J. Mol. Sci..

[88] Chen H., Montagnani M., Funahashi T., Shimomura I., Quon M.J. (2003). Adiponectin stimulates production of nitric oxide in vascular endothelial cells. J. Biol. Chem..

[89] Ouchi N., Kobayashi H., Kihara S., Kumada M., Sato K., Inoue T., Funahashi T., Walsh K. (2004). Adiponectin stimulates angiogenesis by promoting cross-talk between AMP-activated protein kinase and Akt signaling in endothelial cells. J. Biol. Chem..

[90] Cheng K.K., Lam K.S., Wang Y., Huang Y., Carling D., Wu D., Wong C., Xu A. (2007). Adiponectin-induced endothelial nitric oxide synthase activation and nitric oxide production are mediated by APPL1 in endothelial cells. Diabetes.

[91] Philippova M., Joshi M.B., Kyriakakis E., Pfaff D., Erne P., Resink T.J. (2009). A guide and guard: The many faces of T-cadherin. Cell Signal..

[92] Nacci C., Leo V., de Benedictis L., Potenza M.A., Sgarra L., de Salvia M.A., Quon M.J., Montagnani M. (2016). Infliximab therapy restores adiponectin expression in perivascular adipose tissue and improves endothelial nitric oxide-mediated vasodilation in mice with type 1 diabetes. Vascul. Pharmacol..

[93] Lavie C.J., Arena R., Swift D.L., Johannsen N.M., Sui X., Lee D.C., Earnest C.P., Church T.S., O’Keefe J.H., Milani R.V. (2015). Exercise and the cardiovascular system: Clinical science and cardiovascular outcomes. Circ. Res..

[94] Green D.J., Spence A., Halliwill J.R., Cable N.T., Thijssen D.H. (2011). Exercise and vascular adaptation in asymptomatic humans. Exp. Physiol..

[95] Pedersen B.K., Saltin B. (2006). Evidence for prescribing exercise as therapy in chronic disease. Scand. J. Med. Sci. Sports.

[96] Meziat C., Boulghobra D., Strock E., Battault S., Bornard I., Walther G., Reboul C. (2019). Exercise training restores eNOS activation in the perivascular adipose tissue of obese rats: Impact on vascular function. Nitric Oxide.

[97] Cybularz M., Langbein H., Zatschler B., Brunssen C., Deussen A., Matschke K., Morawietz H. (2017). Endothelial function and gene expression in perivascular adipose tissue from internal mammary arteries of obese patients with coronary artery disease. Atheroscler. Suppl..

[98] Baltieri N., Guizoni D.M., Victorio J.A., Davel A.P. (2018). Protective Role of Perivascular Adipose Tissue in Endothelial Dysfunction and Insulin-Induced Vasodilatation of Hypercholesterolemic LDL Receptor-Deficient Mice. Front. Physiol..

[99] Badran M., Yassin B.A., Lin D., Kobor M.S., Ayas N., Laher I. (2019). Gestational intermittent hypoxia induces endothelial dysfunction, reduces perivascular adiponectin and causes epigenetic changes in adult male offspring. J. Physiol..

[100] Almabrouk T., White A.D., Ugusman A.B., Skiba D.S., Katwan O.J., Alganga H., Guzik T.J., Touyz R.M., Salt I.P., Kennedy S. (2018). High Fat Diet Attenuates the Anticontractile Activity of Aortic PVAT via a Mechanism Involving AMPK and Reduced Adiponectin Secretion. Front. Physiol..

[101] Zaborska K.E., Wareing M., Edwards G., Austin C. (2016). Loss of anti-contractile effect of perivascular adipose tissue in offspring of obese rats. Int. J. Obes..

[102] Loria A.S., Spradley F.T., Obi I.E., Becker B.K., de Miguel C., Speed J.S., Pollock D.M., Pollock J.S. (2018). Maternal separation enhances anticontractile perivascular adipose tissue function in male rats on a high-fat diet. Am. J. Physiol. Regul. Integr. Comp. Physiol..

[103] Komura N., Maeda N., Mori T., Kihara S., Nakatsuji H., Hirata A., Tochino Y., Funahashi T., Shimomura I. (2013). Adiponectin protein exists in aortic endothelial cells. PLoS ONE.

[104] Ma Y., Li L., Shao Y., Bai X., Bai T., Huang X. (2017). Methotrexate improves perivascular adipose tissue/endothelial dysfunction via activation of AMPK/eNOS pathway. Mol. Med. Rep..

[105] Han F., Li K., Pan R., Xu W., Han X., Hou N., Sun X. (2018). Calycosin directly improves perivascular adipose tissue dysfunction by upregulating the adiponectin/AMPK/eNOS pathway in obese mice. Food Funct..

[106] Van Dam R.M., Naidoo N., Landberg R. (2013). Dietary flavonoids and the development of type 2 diabetes and cardiovascular diseases: Review of recent findings. Curr. Opin. Lipidol..

[107] Wedick N.M., Pan A., Cassidy A., Rimm E.B., Sampson L., Rosner B., Willett W., Hu F.B., Sun Q., van Dam R.M. (2012). Dietary flavonoid intakes and risk of type 2 diabetes in US men and women. Am. J. Clin. Nutr..

[108] Cassidy A., Mukamal K.J., Liu L., Franz M., Eliassen A.H., Rimm E.B. (2013). High anthocyanin intake is associated with a reduced risk of myocardial infarction in young and middle-aged women. Circulation.

[109] Liu Y., Li D., Zhang Y., Sun R., Xia M. (2014). Anthocyanin increases adiponectin secretion and protects against diabetes-related endothelial dysfunction. Am. J. Physiol. Endocrinol. Metab..

[110] Wang Y., Zhang Y., Wang X., Liu Y., Xia M. (2012). Supplementation with cyanidin-3-O-β-glucoside protects against hypercholesterolemia-mediated endothelial dysfunction and attenuates atherosclerosis in apolipoprotein E-deficient mice. J. Nutr..

[111] Corvera S., Gealekman O. (2013). Adipose tissue angiogenesis: Impact on obesity and type-2 diabetes. Biochim. Biophys. Acta..

[112] Elias I., Franckhauser S., Ferré T., Vilà L., Tafuro S., Muñoz S., Roca C., Ramos D., Pujol A., Riu E. (2012). Adipose tissue overexpression of vascular endothelial growth factor protects against diet-induced obesity and insulin resistance. Diabetes.

[113] Sun K., Wernstedt Asterholm I., Kusminski C.M., Bueno A.C., Wang Z.V., Pollard J.W., Brekken R.A., Scherer P.E. (2012). Dichotomous effects of VEGF-A on adipose tissue dysfunction. Proc. Natl. Acad. Sci. USA.

[114] Sun K., Kusminski C.M., Luby-Phelps K., Spurgin S.B., An Y.A., Wang Q.A., Holland W.L., Scherer P.E. (2014). Brown adipose tissue derived VEGF-A modulates cold tolerance and energy expenditure. Mol. Metab..

[115] Sung H.K., Doh K.O., Son J.E., Park J.G., Bae Y., Choi S., Nelson S.M., Cowling R., Nagy K., Michael I.P. (2013). Adipose vascular endothelial growth factor regulates metabolic homeostasis through angiogenesis. Cell Metab..

[116] Shimizu I., Aprahamian T., Kikuchi R., Shimizu A., Papanicolaou K.N., MacLauchlan S., Maruyama S., Walsh K. (2014). Vascular rarefaction mediates whitening of brown fat in obesity. J. Clin. Investig..

[117] Gharakhanian R., Su S., Aprahamian T. (2019). Vascular Endothelial Growth Factor-A Deficiency in Perivascular Adipose Tissue Impairs Macrovascular Function. Front. Physiol..

[118] Libby P., Ridker P.M., Hansson G.K. (2011). Progress and challenges in translating the biology of atherosclerosis. Nature.

[119] Okamoto Y., Kihara S., Ouchi N., Nishida M., Arita Y., Kumada M., Ohashi K., Sakai N., Shimomura I., Kobayashi H. (2002). Adiponectin reduces atherosclerosis in apolipoprotein E-deficient mice. Circulation.

[120] Wolf A.M., Wolf D., Rumpold H., Enrich B., Tilg H. (2004). Adiponectin induces the anti-inflammatory cytokines IL-10 and IL-1RA in human leukocytes. Biochem. Biophys. Res. Commun..

[121] Ohashi K., Parker J.L., Ouchi N., Higuchi A., Vita J.A., Gokce N., Pedersen A.A., Kalthoff C., Tullin S., Sams A. (2010). Adiponectin promotes macrophage polarization toward an anti-inflammatory phenotype. J. Biol. Chem..

[122] Ouchi N., Kihara S., Arita Y., Nishida M., Matsuyama A., Okamoto Y., Ishigami M., Kuriyama H., Kishida K., Nishizawa H. (2001). Adipocyte-derived plasma protein, adiponectin, suppresses lipid accumulation and class A scavenger receptor expression in human monocyte-derived macrophages. Circulation.

[123] Tian L., Luo N., Klein R.L., Chung B.H., Garvey W.T., Fu Y. (2009). Adiponectin reduces lipid accumulation in macrophage foam cells. Atherosclerosis.

[124] Tian L., Luo N., Zhu X., Chung B.H., Garvey W.T., Fu Y. (2012). Adiponectin-AdipoR1/2-APPL1 signaling axis suppresses human foam cell formation: Differential ability of AdipoR1 and AdipoR2 to regulate inflammatory cytokine responses. Atherosclerosis.

[125] Wang M., Wang D., Zhang Y., Wang X., Liu Y., Xia M. (2013). Adiponectin increases macrophages cholesterol efflux and suppresses foam cell formation in patients with type 2 diabetes mellitus. Atherosclerosis.

[126] Arita Y., Kihara S., Ouchi N., Maeda K., Kuriyama H., Okamoto Y., Kumada M., Hotta K., Nishida M., Takahashi M. (2002). Adipocyte-derived plasma protein adiponectin acts as a platelet-derived growth factor-BB-binding protein and regulates growth factor-induced common postreceptor signal in vascular smooth muscle cell. Circulation.

[127] Ouchi N., Kihara S., Arita Y., Maeda K., Kuriyama H., Okamoto Y., Hotta K., Nishida M., Takahashi M., Nakamura T. (1999). Novel modulator for endothelial adhesion molecules: Adipocyte-derived plasma protein adiponectin. Circulation.

[128] Ouchi N., Kihara S., Arita Y., Okamoto Y., Maeda K., Kuriyama H., Hotta K., Nishida M., Takahashi M., Muraguchi M. (2000). Adiponectin, an adipocyte-derived plasma protein, inhibits endothelial NF-kappaB signaling through a cAMP-dependent pathway. Circulation.

[129] Plant S., Shand B., Elder P., Scott R. (2008). Adiponectin attenuates endothelial dysfunction induced by oxidised low-density lipoproteins. Diab. Vasc. Dis. Res..

[130] Saneipour M., Ghatreh-Samani K., Heydarian E., Farrokhi E., Abdian N. (2015). Adiponectin inhibits oxidized low density lipoprotein-induced increase in matrix metalloproteinase 9 expression in vascular smooth muscle cells. ARYA Atheroscler..

[131] Fang H., Judd R.L. (2018). Adiponectin Regulation and Function. Compr. Physiol..

[132] Kroemer G., Mariño G., Levine B. (2010). Autophagy and the integrated stress response. Mol. Cell..

[133] Peng N., Meng N., Wang S., Zhao F., Zhao J., Su L., Zhang S., Zhang Y., Zhao B., Miao J. (2014). An activator of mTOR inhibits oxLDL-induced autophagy and apoptosis in vascular endothelial cells and restricts atherosclerosis in apolipoprotein E⁻/⁻ mice. Sci. Rep..

[134] Li B.H., Yin Y.W., Liu Y., Pi Y., Guo L., Cao X.J., Gao C.Y., Zhang L.L., Li J.C. (2014). TRPV1 activation impedes foam cell formation by inducing autophagy in oxLDL-treated vascular smooth muscle cells. Cell Death Dis..

[135] Li C., Wang Z., Wang C., Ma Q., Zhao Y. (2015). Perivascular adipose tissue-derived adiponectin inhibits collar-induced carotid atherosclerosis by promoting macrophage autophagy. PLoS ONE.

[136] Gruzdeva O.V., Belik E.V., Dyleva Y.A., Borodkina D.A., Sinitsky M.Y., Naumov D.Y., Bychkova E.E., Fanaskova E.V., Palicheva E.I., Kuzmina A.A. (2021). Expression of adipocytokines in heart fat depots depending on the degree of coronary artery atherosclerosis in patients with coronary artery disease. PLoS ONE.

[137] Villarroya F., Iglesias R., Giralt M. (2004). Retinoids and retinoid receptors in the control of energy balance: Novel pharmacological strategies in obesity and diabetes. Curr. Med. Chem..

[138] Bastien J., Rochette-Egly C. (2004). Nuclear retinoid receptors and the transcription of retinoid-target genes. Gene.

[139] Zhou B., Pan Y., Hu Z., Wang X., Han J., Zhou Q., Zhai Z., Wang Y. (2012). All-trans-retinoic acid ameliorated high fat diet-induced atherosclerosis in rabbits by inhibiting platelet activation and inflammation. J. Biomed. Biotechnol..

[140] Van Y.H., Lee W.H., Ortiz S., Lee M.H., Qin H.J., Liu C.P. (2009). All-trans retinoic acid inhibits type 1 diabetes by T regulatory (Treg)-dependent suppression of interferon-gamma-producing T-cells without affecting Th17 cells. Diabetes.

[141] Kalisz M., Chmielowska M., Martyńska L., Domańska A., Bik W., Litwiniuk A. (2021). All-trans-retinoic acid ameliorates atherosclerosis, promotes perivascular adipose tissue browning, and increases adiponectin production in Apo-E mice. Sci. Rep..

[142] Achari A.E., Jain S.K. (2017). Adiponectin, a Therapeutic Target for Obesity, Diabetes, and Endothelial Dysfunction. Int. J. Mol. Sci..

[143] Phillips S.A., Ciaraldi T.P., Kong A.P., Bandukwala R., Aroda V., Carter L., Baxi S., Mudaliar S.R., Henry R.R. (2003). Modulation of circulating and adipose tissue adiponectin levels by antidiabetic therapy. Diabetes.

[144] Combs T.P., Wagner J.A., Berger J., Doebber T., Wang W.J., Zhang B.B., Tanen M., Berg A.H., O’Rahilly S., Savage D.B. (2002). Induction of adipocyte complement-related protein of 30 kilodaltons by PPARgamma agonists: A potential mechanism of insulin sensitization. Endocrinology.

[145] Kubota N., Terauchi Y., Kubota T., Kumagai H., Itoh S., Satoh H., Yano W., Ogata H., Tokuyama K., Takamoto I. (2006). Pioglitazone ameliorates insulin resistance and diabetes by both adiponectin-dependent and -independent pathways. J. Biol. Chem..

[146] Hernandez A.V., Usmani A., Rajamanickam A., Moheet A. (2011). Thiazolidinediones and risk of heart failure in patients with or at high risk of type 2 diabetes mellitus: A meta-analysis and meta-regression analysis of placebo-controlled randomized clinical trials. Am. J. Cardiovasc. Drugs.

[147] Aronoff S., Rosenblatt S., Braithwaite S., Egan J.W., Mathisen A.L., Schneider R.L. (2000). Pioglitazone hydrochloride monotherapy improves glycemic control in the treatment of patients with type 2 diabetes: A 6-month randomized placebo-controlled dose-response study. The Pioglitazone 001 Study Group. Diabetes Care.

[148] Vestergaard P. (2007). Discrepancies in bone mineral density and fracture risk in patients with type 1 and type 2 diabetes—A meta-analysis. Osteoporos. Int..

[149] Higgins L.S., Mantzoros C.S. (2008). The Development of INT131 as a Selective PPARgamma Modulator: Approach to a Safer Insulin Sensitizer. PPAR Res..

[150] Clasen R., Schupp M., Foryst-Ludwig A., Sprang C., Clemenz M., Krikov M., Thöne-Reineke C., Unger T., Kintscher U. (2005). PPARgamma-activating angiotensin type-1 receptor blockers induce adiponectin. Hypertension.

[151] Watanabe S., Okura T., Kurata M., Irita J., Manabe S., Miyoshi K., Fukuoka T., Murakami K., Higaki J. (2006). The effect of losartan and amlodipine on serum adiponectin in Japanese adults with essential hypertension. Clin. Ther..

[152] Mohammadi A., Gholamhoseinian A., Fallah H. (2014). Zataria multiflora increases insulin sensitivity and PPARγ gene expression in high fructose fed insulin resistant rats. Iran. J. Basic Med. Sci..

[153] Gómez-Arbeláez D., Lahera V., Oubiña P., Valero-Muñoz M., de Las Heras N., Rodríguez Y., García R.G., Camacho P.A., López-Jaramillo P. (2013). Aged garlic extract improves adiponectin levels in subjects with metabolic syndrome: A double-blind, placebo-controlled, randomized, crossover study. Mediators Inflamm..

[154] Jürimäe J., Hofmann P., Jürimäe T., Mäestu J., Purge P., Wonisch M., Pokan R., von Duvillard S.P. (2006). Plasma adiponectin response to sculling exercise at individual anaerobic threshold in college level male rowers. Int. J. Sports Med..

[155] Jürimäe J., Purge P., Jürimäe T. (2005). Adiponectin is altered after maximal exercise in highly trained male rowers. Eur. J. Appl Physiol..

[156] Woodward L., Akoumianakis I., Antoniades C. (2017). Unravelling the adiponectin paradox: Novel roles of adiponectin in the regulation of cardiovascular disease. Br. J. Pharmacol..

[157] Halberg N., Schraw T.D., Wang Z.V., Kim J.Y., Yi J., Hamilton M.P., Luby-Phelps K., Scherer P.E. (2009). Systemic fate of the adipocyte-derived factor adiponectin. Diabetes.

[158] Tullin S., Sams A., Brandt J., Dahl K., Gong W., Jeppesen C.B., Krogh T.N., Olsen G.S., Liu Y., Pedersen A.A. (2012). Recombinant adiponectin does not lower plasma glucose in animal models of type 2 diabetes. PLoS ONE.

[159] Zhao S., Kusminski C.M., Scherer P.E. (2021). Adiponectin, Leptin and Cardiovascular Disorders. Circ. Res..

[160] Tacke F., Wüstefeld T., Horn R., Luedde T., Srinivas Rao A., Manns M.P., Trautwein C., Brabant G. (2005). High adiponectin in chronic liver disease and cholestasis suggests biliary route of adiponectin excretion in vivo. J. Hepatol..

[161] Lee C.H., Lui D.T.W., Cheung C.Y.Y., Fong C.H.Y., Yuen M.M.A., Chow W.S., Woo Y.C., Xu A., Lam K.S.L. (2020). Higher circulating adiponectin concentrations predict incident cancer in type 2 diabetes—The adiponectin paradox. J. Clin. Endocrinol. Metab..

[162] Baker J.F., Newman A.B., Kanaya A., Leonard M.B., Zemel B., Milijkovic I., Long J., Webber D., Harris T.B. (2019). The adiponectin paradox in the elderly: Associations with body composition, physical functioning, and mortality. J. Gerontol. A Biol. Sci. Med. Sci..

[163] Tsutamoto T., Tanaka T., Sakai H., Ishikawa C., Fujii M., Yamamoto T., Horie M. (2007). Total and high molecular weight adiponectin, haemodynamics, and mortality in patients with chronic heart failure. Eur. Heart J..

[164] Karas M.G., Benkeser D., Arnold A.M., Bartz T.M., Djousse L., Mukamal K.J., Ix J.H., Zieman S.J., Siscovick D.S., Tracy R.P. (2014). Relations of plasma total and high-molecular-weight adiponectin to new-onset heart failure in adults ≥65 years of age (from the Cardiovascular Health study). Am. J. Cardiol..

[165] Kim Y., Lim J.H., Kim M.Y., Kim E.N., Yoon H.E., Shin S.J., Choi B.S., Kim Y.S., Chang Y.S., Park C.W. (2018). The Adiponectin Receptor Agonist AdipoRon Ameliorates Diabetic Nephropathy in a Model of Type 2 Diabetes. J. Am. Soc. Nephrol..

[166] Zhang Y., Zhao J., Li R., Lau W.B., Yuan Y.X., Liang B., Li R., Gao E.H., Koch W.J., Ma X.L. (2015). AdipoRon, the first orally active adiponectin receptor activator, attenuates postischemic myocardial apoptosis through both AMPK-mediated and AMPK-independent signalings. AM J. Physiol. Endocrinol. Metab..

[167] Nicolas S., Debayle D., Béchade C., Maroteaux L., Gay A.S., Bayer P., Heurteaux C., Guyon A., Chabry J. (2018). Adiporon, an adiponectin receptor agonist acts as an antidepressant and metabolic regulator in a mouse model of depression. Transl. Psychiatry.

[168] Messaggio F., Mendonsa A.M., Castellanos J., Nagathihalli N.S., Gorden L., Merchant N.B., VanSaun M.N. (2017). Adiponectin receptor agonists inhibit leptin induced pSTAT3 and in vivo pancreatic tumor growth. Oncotarget.

[169] Akimoto M., Maruyama R., Kawabata Y., Tajima Y., Takenaga K. (2018). Antidiabetic adiponectin receptor agonist AdipoRon suppresses tumour growth of pancreatic cancer by inducing RIPK1/ERK-dependent necroptosis. Cell Death Dis..

[170] Hong K., Lee S., Li R., Yang Y., Tanner M.A., Wu J., Hill M.A. (2016). Adiponectin receptor agonist, AdipoRon, causes vasorelaxation predominantly via a direct smooth muscle action. Microcirculation.

[171] Li N., Zhao S., Zhang Z., Zhu Y., Gliniak C.M., Vishvanath L., An Y.A., Wang M.Y., Deng Y., Zhu Q. (2021). Adiponectin preserves metabolic fitness during aging. eLife.

[172] Viljoen A., Sinclair A. (2009). Safety and efficacy of rosiglitazone in the elderly diabetic patient. Vasc. Health Risk Manag..

[173] Yu J.G., Javorschi S., Hevener A.L., Kruszynska Y.T., Norman R.A., Sinha M., Olefsky J.M. (2002). The effect of thiazolidinediones on plasma adiponectin levels in normal, obese, and type 2 diabetic subjects. Diabetes.

